# Examining specific and non-specific symptoms of the best-fitting posttraumatic stress disorder model in conflict-exposed adolescents

**DOI:** 10.1186/s40359-023-01389-8

**Published:** 2023-10-24

**Authors:** Imelu G. Mordeno, Jelli Grace C. Luzano

**Affiliations:** Mindanao State University - Iligan Institute of Technology, Iligan City, Philippines

**Keywords:** Posttraumatic stress disorder, PTSD-specific symptoms, Non-specific PTSD symptoms, Confirmatory factor analysis, Hybrid model

## Abstract

**Background:**

The 5th revision of the Diagnostic and Statistical Manual of Mental Disorders (DSM-5) construes PTSD symptoms into 4 clusters (intrusion, avoidance, negative alterations in cognitions and mood, alterations in arousal and reactivity; Model 1). However, recent literature has shown that this symptom structure does not best represent PTSD. Unfortunately, the findings of studies investigating the proposed alternative models are from consensus. Adding to the complexity of the issue of symptom-grouping models is the identification of specific and non-specific symptoms of PTSD. The present study aims to address these gaps by identifying the best-fitting PTSD model and subsequently examining what symptoms are considered specific and non-specific to PTSD in adolescent-survivors of armed political conflict and violence.

**Methods:**

The study utilized a sample of 641 adolescent victim survivors. We conducted CFA analyses and compared nested models through the scaled χ^2^ difference test, while comparison of non-nested models was done using the Bayesian information criterion (BIC). The best-fitted model was used in the consequent analysis, where we statistically controlled for the effect of non-specific psychological distress on PTSD by comparing the factor loadings and factor correlations before and after accounting for distress using the Aroian z-test.

**Results:**

The results provide support for the 7-factor hybrid model of PTSD over other proposed models for the current sample. Moreover, the data reveal that only 7 items could be construed as core symptoms, while the rest of the symptoms can be considered non-PTSD specific.

**Conclusions:**

Overall, the findings provide support for the validity of the hybrid PTSD model among political conflict-exposed adolescents. The results also show that the DSM-5 PTSD has both specific and non-specific features in the present sample of conflict-exposed adolescents. This has potential implications for theory, practice, and treatment of the disorder.

**Supplementary Information:**

The online version contains supplementary material available at 10.1186/s40359-023-01389-8.

## Background

Several significant changes in the posttraumatic stress disorder (PTSD) symptom criteria have been introduced in the 5th revision of the Diagnostic and Statistical Manual of Mental Disorders (DSM-5) [1]. These changes include the addition (e.g., “reckless/self-destructive behavior”), removal (e.g., “sense of foreshortened future”), and rephrasing (e.g., from “irritability or outbursts of anger” to “irritable behavior and angry outbursts”) of PTSD symptoms. Presently, DSM-5 categorizes PTSD symptoms into intrusion, avoidance, negative alterations in cognition and mood, and alterations of arousal and reactivity symptoms. While the changes in the DSM-5 PTSD addressed the criticisms in previous revisions of the diagnostic criteria, support for the DSM-5 model has been minimal compared to other proposed models [e.g., 2–4]. Adding to the complexity of the issue of symptom-grouping models is the identification of specific and non-specific symptoms of PTSD. These issues have implications for PTSD research and treatment. For instance, empirical support can guide future PTSD research to focus on its salient symptom clusters. Concurrently, information on its specific and non-specific symptoms can inform targeted interventions. Thus, the present study attempts to resolve these concerns by identifying the best-fitting model among existing PTSD models and subsequently examining which symptoms are possibly specific to PTSD or common to other disorders.

More differentiated models have consistently demonstrated superiority over the DSM-5 model (see Table 1). The dysphoria model (Model 2) [5] identifies a unique dysphoria (i.e., general psychological distress) factor which encompasses the DSM-5 model’s negative alterations in cognition and mood and the alterations in arousal and reactivity factors. The dysphoric arousal model (Model 3) [2] divides DSM-5’s alterations in arousal and reactivity factor into dysphoric and anxious arousal factors to distinguish between arousal symptoms that have mixed anxiety and depression features (i.e., dysphoric arousal) and those that are fear-based and more specific to anxiety disorders (i.e., anxious arousal). Building on the dysphoric arousal model, the anhedonia model (Model 4) [3] further distinguishes between negative and positive affect symptoms. This is based on the contention that these two constructs are distinct emotions and warrant separate attention in research and practice [6, 7]. Conversely, the externalizing behaviors model (Model 5) [8] retains the DSM-5’s negative alterations in cognitions and mood factor while highlighting a subset of dysphoric arousal symptoms. It identifies two symptoms of externalized, self-initiated, aggressive behaviors of individuals with PTSD called ‘externalizing behaviors’. Lastly, the hybrid model (Model 6) [4] incorporates the salient features of all previous models, resulting in seven differentiated PTSD symptom clusters (i.e., intrusion, avoidance, negative affect, anhedonia, externalizing behavior, anxious arousal, dysphoric arousal). A summary of the similarities and differences in item grouping for all the models is presented in Table 1.

**Table 1:** Symptom mappings across competing PTSD models

PTSD Symptoms	Model 1 (DSM-5)	Model 2 (DMS − 5 dysphoria)	Model 3 (DSM-5 dysphoric arousal)	Model 4 (Anhedonia)	Model 5 (externalizing behaviors)	Model 6 (hybrid)
B1. Intrusive thoughts	In	In	In	In	In	In
B2. Nightmares	In	In	In	In	In	In
B3. Flashbacks	In	In	In	In	In	In
B4. Emotional Cue Reactivity	In	In	In	In	In	In
B5. Physiological Cue Reactivity	In	In	In	In	In	In
C1. Avoidance of thoughts	Av	Av	Av	Av	Av	Av
C2. Avoidance of reminders	Av	Av	Av	Av	Av	Av
D1. Trauma-related Amnesia	NACM	Dy	NACM	NA	NACM	NA
D2. Negative beliefs	NACM	Dy	NACM	NA	NACM	NA
D3. Distorted blame	NACM	Dy	NACM	NA	NACM	NA
D4. Pervasive Negative Emotional State	NACM	Dy	NACM	NA	NACM	NA
D5. Lack of Interest	NACM	Dy	NACM	An	NACM	An
D6. Feeling detached	NACM	Dy	NACM	An	NACM	An
D7. Inability to experience positive emotions	NACM	Dy	NACM	An	NACM	An
E1. Irritability/Aggression	AAR	Dy	DA	DA	EB	EB
E2. Recklessness	AAR	Dy	DA	DA	EB	EB
E3. Hyper vigilance	AAR	AAR	AA	AA	AA	AA
E4. Exaggerated startle	AAR	AAR	AA	AA	AA	AA
E5. Difficulty concentrating	AAR	Dy	DA	DA	DA	DA
E6. Sleep Disturbance	AAR	Dy	DA	DA	DA	DA

Note: PTSD, Post-Traumatic Stress Disorder ; In, intrusion; Av, avoidance; NACM, negative alterations in cognitions and mood; AAR, alterations in arousal and reactivity; Dy, dysphoria; DA, dysphoric arousal; AA, anxious arousal; EB, externalizing behavior; NA, negative affect; An, anhedonia

A second issue emerging from the changes in DSM-5 PTSD symptomatology pertains to whether the symptoms are disorder-specific or non-specific. Several studies have found that PTSD contains several non-specific symptoms [7, 9] representing general distress [10]. These non-specific distress symptoms appear to be common across mental health disorders [see 5, 7, 11]. However, information pertaining to which specific symptoms or symptom clusters are more related to PTSD than general distress remains scant. Identifying disorder-specific and non-specific symptoms of PTSD has important implications. First, identifying non-specific symptoms of PTSD (e.g., sleep disturbance, difficulty concentrating) is important for understanding patients’ clinical presentation, degree of impairment, and probable response to different treatment modalities. Numerous studies have shown that the presence of comorbid non-specific disorder symptoms is associated with more severe PTSD and worse functionality [12, 13]. Impliedly, identifying the non-specific symptoms of PTSD can inform clinicians and mental health practitioners on the pervasiveness of traumatic events’ impact, allowing for the implementation of effective interventions [14, 15]. Second, it is equally important to identify PTSD-specific symptoms. PTSD as a diagnostic entity is presumed to have cardinal symptoms unique from other disorders. Identifying these core symptoms could lead to a more accurate understanding of PTSD and its distinctiveness from other disorders. Finally, differentiating between specific and non-specific symptoms has pragmatic treatment implications. Emerging studies show that targeting non-specific aspects can reduce overall PTSD severity [e.g., 16]. This may inform a two-pronged approach to the treatment of PTSD: the first arm can address non-specific symptoms to decrease overall symptom severity, while the second focuses on targeting disorder-specific symptoms to alleviate PTSD.

## Method

### Procedure and participants

The present study aims to identify the best-fitting factor structure of PTSD in the current sample and determine PTSD’s specific and non-specific symptom clusters. Prior to data gathering, we sought permission from the appropriate authorities to conduct the data gathering (i.e., division superintendent and principals of the schools). We used purposive sampling to gather data from 684 adolescents living in a province in Southern Philippines, with ‘exposure to armed political conflict and violence’ as the sole inclusion criteria. The participant’s responses on the Exposure to Conflict and Violence Scale determined exposure to trauma events and were validated through interviews with teachers and school counselors. Of the 684, we confirmed that only 641 participants have been exposed to political conflict and violence. After obtaining assent forms from the participants and informed consent forms from their parents, we invited those who met the inclusion criteria to participate in the study.

The present sample is a community sample of 641 adolescents exposed to armed political conflict and violence. All participants reside in a province in Southern Philippines. The sample is predominantly female (n = 416, 64.9%), between ages 13–17 (M = 16.47, SD = 0.89), and Muslim (n = 597, 93.1%). The frequency distribution for the exposure of trauma events is included in Supplementary Table 1. The study’s procedures adhere to the tenets of the Declaration of Helsinki and are reviewed and approved by an ethics committee from the Mindanao State University – Iligan Institute of Technology College of Education, Philippines.

### Instruments

*Exposure to political conflict and violence.* Participants indicate exposure to conflict and violence on a 29-item questionnaire enumerating the most common war experiences of the people in the vicinity. The first 28 items list the event (e.g., loss of, or injury to, a friend or family member; witnessed actual violence such as seeing a stranger being arrested, injured, or killed; violence among groups of people or the population generally that took place in neighborhoods, streets, and around checkpoints that people crossed as they sought to reach work, schooling and services). One additional item allows the participant to identify experiences not included in the first 28 items. Participants indicate whether or not they experienced any of the events listed in the past year, and how often they have experienced them, using a four-point scale (0=“not at all/never” to 4=“many times/almost everyday”). The scale has a Cronbach alpha of 0.92.

*Posttraumatic stress symptoms.* The PTSD Checklist for DSM-5 (PCL-5) [17] is a 20-item self-report measure used to assess PTSD symptoms within the last six months. PCL-5 is based on the DSM-5 criteria of PTSD comprising four factors: intrusion (e.g., “Repeated, disturbing, and unwanted memories of the stressful experience”), avoidance (e.g., “Avoiding memories, thoughts, or feelings related to the stressful experience”), negative alterations in cognition and mood (e.g., “Having strong negative feelings such as fear, horror, anger, guilt, or shame”), and alterations in arousal and reactivity (e.g., “Being ‘super alert’ or ‘watchful or on guard’”). Participants rate each item on a five-point Likert scale ranging from 0 (“Not at all”) to 4 (“Extremely”), based on their experience of evacuating during armed conflicts. Research shows that PCL-5 is valid and reliable [e.g., 3, 18], even among Filipinos [e.g., 19–21]. In the present study, the PCL-5 has a Cronbach alpha of 0.92.

*Psychological distress.* Psychological distress is measured using the Kessler Psychological Distress Scale (K10) [22]. The K10 consists of 10 items describing an individual’s experience of anxiety and depression symptoms within the last 30 days (e.g., “About how often did you feel tired out for no good reason”, “About how often did you feel nervous”, “About how often did you feel so nervous that nothing could calm you down”). They rate each item on a five-point Likert scale ranging from 1 (“None of the time”) to 5 (“All of the time”). Research shows the K10 to have good reliability and validity [e.g., 22, 23]. In the present study, the scale has a Cronbach alpha of 0.87.

### Data analysis

Prior to any statistical analyses, we screened the data and found that values were missing completely at random (MCAR). For the K10, 40 (6.2%) participants missed 1 item, 11 (1.7%) were missing 2 items, and 47 (7.3%) were missing 3 items. For the PCL-5, 107 (16.7%) participants missed 1 item, 32 (5.0%) were missing 2 items, 7 (1.1%) were missing 3 items, 2 (0.3%) were missing 4 items, 1 (0.2%) were missing 5 items, and 28 (4.4%) were missing 7 items. We used expectation-maximization (EM) [24], as EM methods have shown effectiveness in treating missing data [62] for up to 30% missing values [25]. None of the respondents were excluded from the analysis based on missing responses. We conducted CFAs using robust maximum likelihood estimation method with mean-adjusted Satorra-Bentler chi-square (S-Bχ^2^) to correct for non-normality. In all CFA analyses, all factors were allowed to correlate but correlated errors were not permitted.

Comparison of nested models was done using the scaled χ^2^ difference test [26], while comparison of non-nested models was done using the Bayesian information criterion (BIC). Next, we created a combined PTSD-Psychological Distress model and regressed the items of the best-fitted PTSD model to the total psychological distress score, allowing us to statistically control for the effect of non-specific psychological distress on PTSD. We consequently compared the factor loadings and factor correlations before and after including psychological distress using the Aroian z-test [27]. This test helped us determine whether there is a significant change in PTSD symptoms’ factor loadings once psychological distress is controlled for.

Assessing the significant changes in PTSD symptoms’ factor loadings before and after controlling for psychological distress helped to distinguish between PTSD-specific and non-specific symptoms. This was based on the notion that when psychological distress is statistically accounted for, the factor loadings of PTSD item-symptoms will significantly decrease particularly for those with substantial associations (i.e., correlations) with psychological distress. We considered these item-symptoms that have significant shared variance with general distress as non-specific PTSD symptoms. Meanwhile, PTSD item-symptoms whose factor loadings did not significantly attenuate may have less substantial relationships with psychological distress. Given that they did not share a significant amount of variance, these symptoms were considered PTSD-specific. Several studies have utilized this approach to assess whether the symptoms were unique to PTSD or shared with other disorders [e.g., 28–30]. To correct for Type I error, the Bonferroni-Holm method was used [31]. All analyses were conducted using the Mplus software version 7.11 [32].

## Results

The current sample has a mean score of 26.04 (SD = 13.40) on the PCL, with participants scoring between 0 and 80. Meanwhile, the mean score for K10 is 13.05 (SD = 7.07), with participants scoring between 10 and 47. Results of the CFA are summarized in Table 2. All six models achieved adequate to excellent fit based on CFI, TLI and RMSEA values. Comparison of nested and non-nested models are summarized in Tables 2 and 3. Among the models, model 6 (hybrid model) shows the best fit to the data (S-Bχ^2^ (149, N = 641) = 270.061, p < 0.0001, CFI = 0.967, TLI = 0.958, RMSEA = 0.036 (C.I.=0.029–0.042)). Model 3 (dysphoric arousal model) achieves better fit than models 1 (DSM-5 model) and 2 (dysphoria model), but not models 4 (anhedonia model), 5 (externalizing behaviors model), and 6.

**Table 2:** Model goodness-of-fit indices (MLM)

Models	S-B χ²	*df*	CFI	TLI	RMSEA	RMSEA 90%CI	BIC
1	395.667	164	0.937	0.927	0.047	0.041 0.053	563.706
2	390.820	164	0.938	0.928	0.046	0.041 0.052	558.859
3	376.309	160	0.941	0.930	0.046	0.040 0.052	570.200
4	327.000	155	0.953	0.942	0.042	0.035 0.048	553.206
5	327.444	155	0.953	0.942	0.042	0.035 0.048	553.650
6	270.061	149	0.967	0.958	0.036	0.029 0.042	535.045

Note: Model 1 = DSM-5 model; Model 2 = DSM-5 dysphoria model; Model 3 = DSM-5 dysphoric arousal model; Model 4 = Anhedonia model; Model 5 = externalizing behaviors model; Model 6 = Hybrid model; CFI, Comparative fit index; TLI, Tucker-Lewis index; BIC, Bayesian Information Criterion; RMSEA, root mean square error of approximation; CI, confidence interval. N = 641

**Fig. 1:**
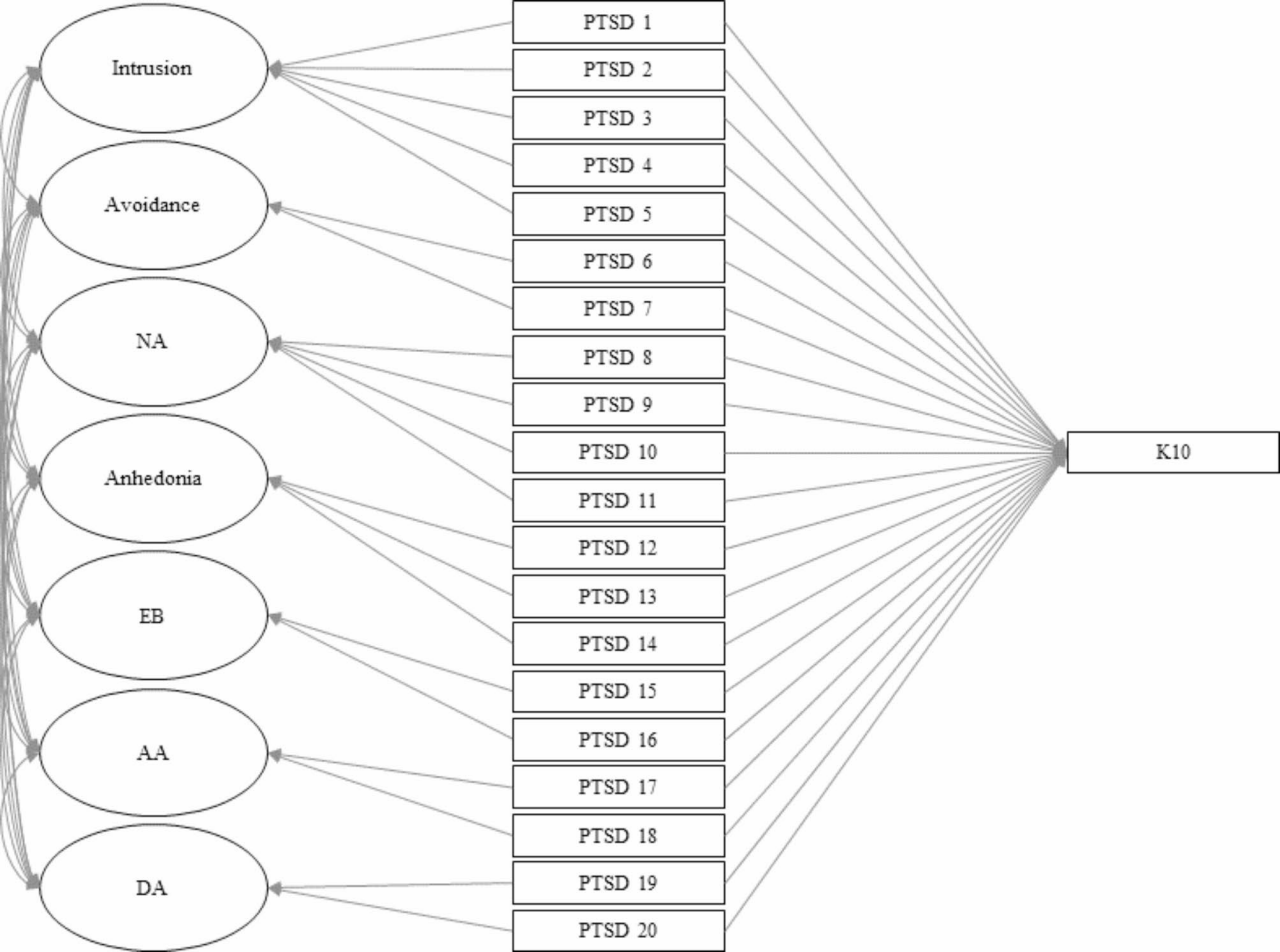
The combined PTSD-Psychological Distress model

Given these findings, we used the hybrid model in the consequent analysis. We regressed the hybrid model’s items to the observed K10 total score (see Fig. 1). The combined PTSD-Psychological Distress model also has excellent fit (S-Bχ^2^ (149, N = 641) = 272.016, p < 0.0001, CFI = 0.969, TLI = 0.956, RMSEA = 0.036 (C.I.=0.029–0.043)). After using the Bonferroni-Holm correction, the decrease in factor loadings for the following items are statistically significant: B2, nightmares; B5, physiological cue reactivity; C2, avoidance of reminders; D1, trauma-related amnesia; D2, negative beliefs; D3, distorted blame; D4, pervasive negative emotional state; D5, lack of interest; D6, detachment; D7, inability to experience positive emotions; E2, recklessness; E5, difficulty concentrating, and; E6, sleep disturbance. Results of this analysis are summarized in Table 3.

**Table 3:** Factor loadings with Aroian z-test values

Symptom		r_1_ (r_2_)	z-value (p)	r_K10_
In	B1. Intrusive thoughts	0.515 (0.412)	2.349 (0.019)*	0.306
In	B2. Nightmares	0.709 (0.587)	3.788 (0.000)**	0.396
In	B3. Flashbacks	0.679 (0.609)	2.142 (0.032)*	0.315
In	B4. Emotional cue reactivity	0.694 (0.611)	2.592 (0.010)*	0.336
In	B5. Physiological cue reactivity	0.657 (0.546)	3.123 (0.002)**	0.363
Av	C1. Avoidance of thoughts	0.708 (0.619)	2.854 (0.004)*	0.349
Av	C2. Avoidance of reminders	0.754 (0.643)	3.909 (0.000)**	0.387
NA	D1. Trauma-related amnesia	0.657 (0.539)	3.300 (0.001)**	0.378
NA	D2. Negative beliefs	0.643 (0.522)	3.290 (0.001)**	0.377
NA	D3. Distorted blame	0.706 (0.567)	4.216 (0.000)**	0.419
NA	D4. Pervasive negative emotional state	0.654 (0.529)	3.456 (0.001)**	0.383
An	D5. Lack of interest	0.709 (0.587)	3.788 (0.000)**	0.396
An	D6. Detachment	0.696 (0.584)	3.411 (0.000)**	0.378
An	D7. Inability to experience positive emotions	0.748 (0.643)	3.664 (0.000)**	0.387
EB	E1. Irritability/Anger	0.715 (0.624)	2.961 (0.003)*	0.355
EB	E2. Recklessness	0.682 (0.569)	3.336 (0.001)**	0.373
AA	E3. Hypervigilance	0.556 (0.476)	1.951 (0.051)	0.280
AA	E4. Exaggerated startle response	0.773 (0.650)	2.826 (0.004)*	0.348
DA	E5. Difficulty concentrating	0.749 (0.612)	4.618 (0.000)**	0.439
DA	E6. Sleep disturbance	0.654 (0.484)	4.260 (0.000)**	0.426

Note: In, intrusion; Av, avoidance; NA, negative affect; An, anhedonia; EB, externalizing behavior; AA, anxious arousal; DA, dysphoric arousal; r_1_ = factor loading of PTSD item to its respective PTSD cluster prior to controlling for psychological distress; r_2_ = factor loading of PTSD item to its respective PTSD cluster after controlling for psychological distress; r_K10_=factor loading of psychological distress variable to each PTSD item

* p-value significant at 0.05

** p-value significant at 0.0025

## Discussion

The present study aims to address two objectives: first, to compare existing PTSD models, and identify the best-fitting model in a political conflict-exposed adolescent sample; and second, to examine which symptoms are PTSD-specific or non-specific. Our results indicate that the best-fitting model is the seven-factor hybrid model of PTSD [4]. The hybrid model has received particularly strong support in recent years across various samples [e.g., 20, 33]. Our results extend support for the model and contend that the hybrid model best represents the PTSD symptom structure in conflict-exposed adolescents. The hybrid model identifies seven distinct factors of PTSD: intrusion, avoidance, negative affect, anhedonia, anxious arousal, dysphoric arousal, and externalizing behaviors. A synthesis of previous models, the hybrid model incorporates three salient changes to the DSM-5 PTSD model. First, it split the negative alterations in cognition and mood factor into negative affect and anhedonia. This is based on previous theoretical and empirical evidence that positive and negative affect are distinct constructs [6]. This distinction suggests that trauma survivors’ experience of negative emotions does not necessarily imply reduced experience of positive emotions. Alternatively, the lack of positive emotions (i.e., anhedonia) does not necessarily mean they will experience negative affect (i.e., anger, guilt). Recent CFA literature supports separating the negative affect factor from the anhedonia factor [e.g., 30]. Second, the hybrid model differentiates the dysphoric arousal factor from the anxious arousal factor [2]. This is based on the notion that anxious arousal represents fear-based symptoms more specific to anxiety disorders, while dysphoric arousal represents mixed anxiety and depression indicators of arousal [2]. Differentiating dysphoric arousal and anxious arousal factors has received substantial support from previous studies [e.g., 34, 35], particularly among Asians [e.g., 36, 37] and Filipinos [e.g., 20, 30]. Lastly, the hybrid model identifies a unique externalizing behaviors factor of PTSD. Researchers contend that externalized, self-initiated aggressive behaviors are distinct from other passive internalized symptoms described by dysphoric arousal and anxious arousal factors [8, 38].

The second objective of this study is to identify which symptoms are potentially specific and non-specific. Non-specific symptoms are associated with general distress [16]. In the present study, we assumed that these symptoms are more correlated to psychological distress than PTSD. Thus, symptoms whose relationships with PTSD did not significantly decrease after controlling for psychological distress are assumed to be PTSD-specific. Alternatively, symptoms whose association with PTSD significantly decreased after accounting for distress are assumed to be non-specific to PTSD. There are several noteworthy findings. First, all item loadings in three prominent PTSD symptom groups (i.e., negative affect, anhedonia, and dysphoric arousal) consistently attenuated when psychological distress was accounted for. This suggests that they are more associated with non-specific distress than PTSD factors. These results are consistent with extant literature indicating that negative affect, anhedonia, and dysphoric arousal symptoms are also present in other mental health disorders. For instance, negative affect is also present in depression [39], generalized anxiety disorder [40], addiction problems [41], and eating disorders [42], while anhedonia is associated with anxiety [43], depression [44], and obsessive-compulsive disorder [45]. Moreover, dysphoric arousal symptoms are significantly associated with anxiety [46], depression [47], and substance use disorders [48]. This is in line with Simms and colleagues’ contention that PTSD contains a large dysphoria (i.e., general distress) component representing shared symptoms between PTSD, anxiety, and depression [5]. This dysphoria factor encompasses all three symptom groups identified as non-specific in the present study. This finding may warrant further investigation.

The second salient finding evolves around the symptoms which are not significantly accounted for by general distress. The data shows that factor loadings of anxious arousal cluster symptoms (i.e., hypervigilance, exaggerated startle response) did not significantly decrease after accounting for distress. Anxious arousal symptoms are associated with responses to a feared stimulus—a clear indication of anxiety—wherein a person with PTSD may show anxious reactions to those which remind them of a traumatic event. This in contrast to dysphoric arousal symptoms wherein people with PTSD may show not only symptoms of anxiety but also depression. Potentially, anxious arousal symptoms are unique features of PTSD while dysphoric arousal is more representative of general distress [e.g., 3, 4, 9, 49]. While more research is needed to support these assertions, our results further strengthen the argument that the negative alterations in cognition and mood cluster in DSM-5 contains separate factors of anxious arousal characterized by fear-based symptoms and dysphoric arousal factor that is comprised of anxiety- and depression-related symptoms [2].

Finally, it is theoretically and empirically interesting that PTSD symptom-clusters did not show the expected differential relationships with general distress at the symptom level. For instance, the intrusion symptom-cluster is widely considered a PTSD-specific factor [e.g., 50, 51]. However, only three (of five) symptoms in this cluster did not significantly attenuate after controlling for psychological distress. Similarly, only avoidance of thoughts (C1) from the avoidance cluster and irritability/anger (E1) from the externalizing behaviors cluster show stronger relations to PTSD after controlling for psychological distress. This suggests that PTSD’s symptom clusters are neither purely PTSD-specific nor non-specific; rather, the heterogeneity of PTSD is present up to the symptom level. This contention is relevant amid the debate on the diagnostic specificity of PTSD. The DSM-5 nomenclature adopts the notion that the wide-ranging consequences of trauma may include symptoms that PTSD shares with other disorders, while the International Classification of Diseases – 11th revision (ICD-11) supports the exclusion of shared symptoms to improve PTSD’s diagnostic specificity and parsimony [9, 52]. Previous research that has aimed to differentiate between specific and non-specific aspects of PTSD anchor their arguments on cluster-level investigations [e.g., 9, 18, 28]. However, the results suggest that such a distinction should consider not just clusters but individual symptoms as well. This does not imply the exclusion of any symptom, however, nor does it advocate for determining diagnoses as such. Simply, the results suggest that the debate regarding PTSD’s nomenclature is far from over, and that looking more closely at individual symptoms may provide a more nuanced picture of its unique properties.

Despite the present study’s contribution to the extant literature, we also recognize its limitations. First, the current sample is a community sample of adolescent-survivors of armed conflict and violence which does not demographically represent Filipino adolescents. Thus, the results are generalizable only to this subset of the population, and any interpretation outside this population must be treated cautiously. It would be interesting if future studies could assess the latent structure of PTSD in minority and marginalized groups, particularly among those who experienced oppression-related distress [53], race-based traumatic stress [54], and internal displacement adversities [21]. Since trauma type is inextricably related to PTSD severity and symptom presentation [47, 53, 55], further investigation on these topics may provide vital information in the etiology, progress, and course of PTSD. The similarities and differences in the symptom structure would substantially contribute to the trauma literature in these least studied populations. Second, this study uses self-report scales. All potential biases and pitfalls of this data-collection approach need to be noted. We also note that because we only use a single measure of psychological distress (i.e., K10), our findings may differ from those which used specific measures of psychological distress, such as anxiety, depression, or both. Further studies investigating PTSD’s unique and transdiagnostic symptoms may benefit from investigating PTSD alongside other diagnostic entities (e.g., generalized anxiety disorder, major depressive disorder) rather than general distress alone. Similarly, future research may look at the impact of other relevant variables. For example, gender appears to affect PTSD symptomatology among adolescents [e.g., 56, 57]. Though we did not find any model invariance across genders (see Supplementary Table 2), it would be interesting to investigate whether gender differences result in model invariances in PTSD, including its differential relations with psychological distress. Third, while the current findings seemingly distinguished between specific and non-specific PTSD symptoms, establishing this distinction require further research as the boundary between PTSD and other disorders is observed to be fuzzy. Fourth, while we observed significant attenuation in several PTSD symptoms after controlling for general distress, it is possible that the relationship between the symptoms may have directly or indirectly affected this decrease. This alternative possibility is beyond the scope of the present study and may be investigated in future research. Fifth, the present study recognizes the importance of utilizing a clinical sample in investigating the factor structure of PTSD. While the present study’s use of a community sample addresses a common caveat of using clinical sample—that is, the lack of generalizability in its findings, particularly to samples who have clinical subthreshold or to those who are still developing symptoms a month after the trauma event—we contend that using clinical samples may provide a more complete picture of the structure, patterns, relationships, and course development of PTSD symptoms from the onset of the trauma event to the later stages of the survivors’ responses and ways of coping. Finally, this is a cross-sectional study. Thus, we recommend conducting longitudinal studies to ascertain the stability of the hybrid model as it relates to psychological distress.

## Conclusions

Amid these limitations, our study has two salient findings that provide important information to enrich the existing trauma literature. First, the findings support the validity of the hybrid model [4] over other PTSD models for the present political conflict-exposed adolescent sample. Given that the hybrid model highlights more specific symptom-factors, this has substantial implications in assessing more precise, multi-factorial PTSD symptomatology and in the development of differentiated and effective interventions targeting these more particular factors. For instance, relaxation exercises (i.e., progressive relaxation) that have been proven effective in decreasing anxiety [58] could be incorporated in lowering the symptom severity of the dysphoric arousal factor, while enhancing social interactions and physical exercise that have been shown to improve positive mood [e.g., 59, 60] could be used to reduce the symptoms of the anhedonia factor. Second, with the results showing different models to be superior to the DSM-5 PTSD model for the current sample, this study advocates for the continued efforts in refining the DSM-5 PTSD configuration and further investigating it across various samples. This may entail further investigation of newer PTSD models such as recently proposed bifactor models [e.g., 35] or revisiting older ones. For instance, our findings suggest that intrusion, avoidance, and anxious arousal symptoms appear to be most central to PTSD in the current sample. This configuration is reminiscent of the earlier three-factor model proposed by King and colleagues [61] composed of intrusion, avoidance, and hyperarousal. Clearly, previous PTSD models may also warrant re-examination. Our second major finding also affirms the previous literature’s observation that DSM-5 PTSD has both specific and non-specific features [e.g., 5, 9]. However, unlike past studies that identify, classify, include, or exclude non-specific symptoms based on clusters and not on specific symptoms, the current data shows that the pattern of non-specific symptoms present in PTSD is not only by clusters (i.e., negative affect, dysphoric arousal, anhedonia) but also on individual symptoms (for example, B2 and B5 symptoms of intrusion cluster are potentially non-specific). The current findings of the study, among other studies [e.g., 9, 18, 20, 28], provide additional information on the symptoms of PTSD, should DSM decide on future revisions of PTSD symptom clusters.

### Electronic supplementary material

Below is the link to the electronic supplementary material.

Supplementary Material 1

## Data Availability

The datasets used and/or analysed during the current study are available from the corresponding author on reasonable request.

## Abbreviations

BIC: Bayesian information criterion
CFA: Confirmatory Factor Analysis
CFI: Comparative fit index
DSM: 5–Diagnostic and Statistical Manual of Mental Disorders 5th revision
EM: Expectation Maximization
ICD: 11–International Classification of Diseases–11th revision
K10: Kessler Psychological Distress Scale 10
NA: Negative affect
PCL: 5–PTSD Checklist for DSM–5
PTSD: Posttraumatic stress disorder
RMSEA: Root mean square error of approximation
TLI: Tucker–Lewis index
