# Supplementary material for: Examining specific and non-specific symptoms of the best-fitting posttraumatic stress disorder model in conflict-exposed adolescents
Source: BMC Psychol. 2023 Oct 24;11:353. doi: 10.1186/s40359-023-01389-8 (PMC10594924; doi:10.1186/s40359-023-01389-8)
Supplement: Supplementary file 1 — Supplementary Material 1 [file 40359_2023_1389_MOESM1_ESM.docx]

| Supplementary Material 1. Frequency distribution of trauma events endorsed by adolescents exposed to armed political conflict and violence (N=641) | | | |
| --- | --- | --- | --- |
| **Trauma event** | **Freq. (%)** | **M** | **SD** |
| Witnessed soldiers in the streets | 567(88.46%) | 1.56 | 0.817 |
| Heard the sounds of bombing | 560(87.36%) | 1.22 | 0.737 |
| Stopped at checkpoints | 516(80.5%) | 1.24 | 0.768 |
| Shooting | 482(75.2%) | 1.14 | 0.791 |
| Violent fights in the street/village, combat situation, or armed conflict that resulted in serious injuries and loss of lives | 157(24.49%) | 0.94 | 0.730 |
| Caught in a crossfire between the paramilitary group and the army | 154(24.02%) | 0.94 | 0.762 |
| Bombing | 150(23.4%) | 0.95 | 0.744 |
| Feelings of not being safe | 149(23.24%) | 0.9 | 0.738 |
| Fire or loud explosion | 140(21.84%) | 0.92 | 0.791 |
| Lack of shelter or not having a home due to forced evacuation | 122(19.03%) | 0.78 | 0.727 |
| Attempted or actual killing of somebody who is not related to you and whom you are not acquainted with | 112(17.47%) | 0.76 | 0.812 |
| Physically harmed, attacked, or beateN up by somebody that has caused physical injury | 111(17.32%) | 0.78 | 0.738 |
| Bereaved | 108(16.85%) | 0.76 | 0.679 |
| Threatened by paramilitary group or members of non-government armed group(s) | 106(16.54%) | 0.86 | 0.788 |
| House broken into | 97(15.13%) | 0.58 | 0.655 |
| In a situation where you could have been seriously injured or killed | 96(14.98%) | 0.70 | 0.694 |
| Not having enough food, water, clothing | 77(12.01%) | 0.86 | 0.722 |
| Threatened by security forces | 75(11.7%) | 0.59 | 0.663 |
| Situation wherein your family, relatives, friends, or anybody close to you got physically harmed, attacked, or beaten up by somebody that has caused physical injury | 74(11.54%) | 0.60 | 0.651 |
| Being told or threatened to be hurt badly, severely injured, or killed by somebody | 72(11.23%) | 0.60 | 0.668 |
| Threatened by a paramilitary group | 70(10.92%) | 0.57 | 0.661 |
| Forced separation from family members | 69(10.76%) | 0.56 | 0.679 |
| House occupied with you in it or thrown out | 65(10.14%) | 0.43 | 0.537 |
| Attempted or actual killing of family members, relatives, friends, or anybody close to you | 48(7.49%) | 0.31 | 0.524 |
| Held at detention | 43(6.71%) | 0.47 | 0.595 |
| Used as a human shield | 42(6.55%) | 0.44 | 0.606 |
| Left alone for many days without any food or anyone taking care of you | 41(6.4%) | 0.55 | 0.620 |
| Death or somebody close to you as a result of suicide | 33(5.15%) | 0.37 | 0.580 |

| Supplementary Table 2. Goodness-of-fit of the Models for Measurement Invariance across Gender groups | | | | | | |
| --- | --- | --- | --- | --- | --- | --- |
| Factor models | Goodness-of-fit indices | | | | Comparison of nested models | |
|  | *S-Bχ^2^* | *df* | CFI | RMSEA | *∆ S-Bχ^2^* | *∆df* |
| Model A | 619.457** | 298 | 0.938 | 0.058 | – | – |
| Model B | 628.424** | 311 | 0.939 | 0.056 | 8.968 | 13 |
| Model C | 649.805** | 324 | 0.937 | 0.056 | 21.381 | 13 |
| *Note:* Model A: Configural invariance (no constraint); Model B: Metric invariance (factor loadings constrained to be equal; Model C: Scalar invariance (intercepts and factor loadings constrained to be equal); *S-Bχ^2^*, Satorra-Bentler chi-square; *df*, degrees of freedom; CFI = comparative fit index; RMSEA = root-mean-square error of approximation; *∆S-Bχ^2^*, change in Satorra-Bentler chi-square using scale correction factor; *∆df*, change in degrees of freedom; **p* < .05, ***p* < .01. | | | | | | |
